# Small-World Propensity Reveals the Frequency Specificity of Resting State Networks

**DOI:** 10.1109/OJEMB.2020.2965323

**Published:** 2020-02-14

**Authors:** Riccardo Iandolo, Marianna Semprini, Stefano Buccelli, Federico Barban, Mingqi Zhao, Jessica Samogin, Gaia Bonassi, Laura Avanzino, Dante Mantini, Michela Chiappalone

**Affiliations:** Rehab TechnologiesIstituto Italiano di Tecnologia 121451 16163 Genova Italy; Rehab TechnologiesIstituto Italiano di Tecnologia 121451 16163 Genova Italy; Department of Informatics, Bioengineering, Robotics and systems Engineering (DIBRIS)University of Genova 504688 Genova Italy; Research Center for Motor Control and NeuroplasticityKatholieke Universiteit Leuven 26657 3001 Leuven Belgium; Department of Experimental Medicine, Section of Human PhysiologyUniversity of Genova 504688 16132 Genova Italy; Department of Experimental Medicine, Section of Human PhysiologyUniversity of Genova 504688 16132 Genova Italy; IRCCS San Martino Hospital 9246 16132 Genova Italy; Research Center for Motor Control and NeuroplasticityKatholieke Universiteit Leuven 26657 3001 Leuven Belgium; IRCSS San Camillo Hospital 30126 Venice Lido Italy

**Keywords:** EEG, frequency specificity, functional connectivity, resting state, small-worldness

## Abstract

*Goal:* Functional connectivity (FC) is an important indicator of the brain's state in different conditions, such as rest/task or health/pathology. Here we used high-density electroencephalography coupled to source reconstruction to assess frequency-specific changes of FC during resting state. Specifically, we computed the Small-World Propensity (SWP) index to characterize network small-world architecture across frequencies. *Methods:* We collected resting state data from healthy participants and built connectivity matrices maintaining the heterogeneity of connection strengths. For a subsample of participants, we also investigated whether the SWP captured FC changes after the execution of a working memory (WM) task. *Results:* We found that SWP demonstrated a selective increase in the alpha and low beta bands. Moreover, SWP was modulated by a cognitive task and showed increased values in the bands entrained by the WM task. *Conclusions:* SWP is a valid metric to characterize the frequency-specific behavior of resting state networks.

## I. Introduction

Human brain imaging by means of non-invasive electrophysiological recordings is a fast-growing field. Recent studies validated the effectiveness of high-density electroencephalography (hdEEG) coupled to source imaging techniques to reconstruct both brain networks during Resting State (RS) [1]–[3] and the activity of deep subcortical areas [4]. Thus, the combination of hdEEG and source imaging allows reliably investigating brain functional connectivity (FC) at both high temporal resolution and good spatial scale, and exploring the frequency-specific properties of functional and effective connectivity structures [5]–[9]. Indeed, each brain rhythm is differently involved in, and therefore associated to, the brain's states of activity (e.g., sleep, rest, tasks) [10]. While the frequency properties of task-related brain activity have been studied [11], [12], frequency-specific FC at rest is still under investigation. It is thus important to define what are the features of neuronal oscillations in whole brain RS networks and to explore the frequency-specific changes induced by a task [2]. Moreover, since oscillations in different frequency bands were demonstrated to play a major role in FC within RSNs [13]–[15], it might be of clinical relevance understanding if RS frequency-specificity could be used as a biomarker for brain pathologies.

Within this framework, here we investigated the frequency-specific properties of FC both at rest and upon a cognitive task. To achieve this, we explored the connectivity strength (local network property) and the small-worldness (global network property) across frequencies, in a cohort of healthy participants. Small-worldness is a widely investigated topological property of human brain networks [16], [17] because it well addresses the integration capabilities (short path length) of multiple highly segregated areas (high clustering). As reported in the FC literature [18], there is a need of metrics that do not depend on graph density and that are estimated from weighted connectivity matrices. To address both requirements, the Small-World Propensity (SWP) was recently proposed, being it unbiased to edge density and specifically defined for use in weighted networks [19]. So far, SWP has been computed primarily from functional and/or structural connectivity estimated with Magnetic Resonance Imaging (MRI) [20]–[24] and with low density EEG montages [25] or magnetoencephalography (MEG) [26]. Other works investigated the frequency-specific properties of FC graphs, however they used metrics different from SWP [8], [27]–[29]. Therefore, to the best of our knowledge, our work is the first employing SWP coupled to hdEEG source imaging and weighted network analysis to highlight FC frequency-specific behavior.

In summary, our *aim* is to *first* evaluate the frequency specificity of FC during RS, as measured by the recent SWP index, and *secondly* to determine whether a cognitive task affects the SWP frequency-specific properties in the following RS session.

## II. Methods

**Fig. 1.**
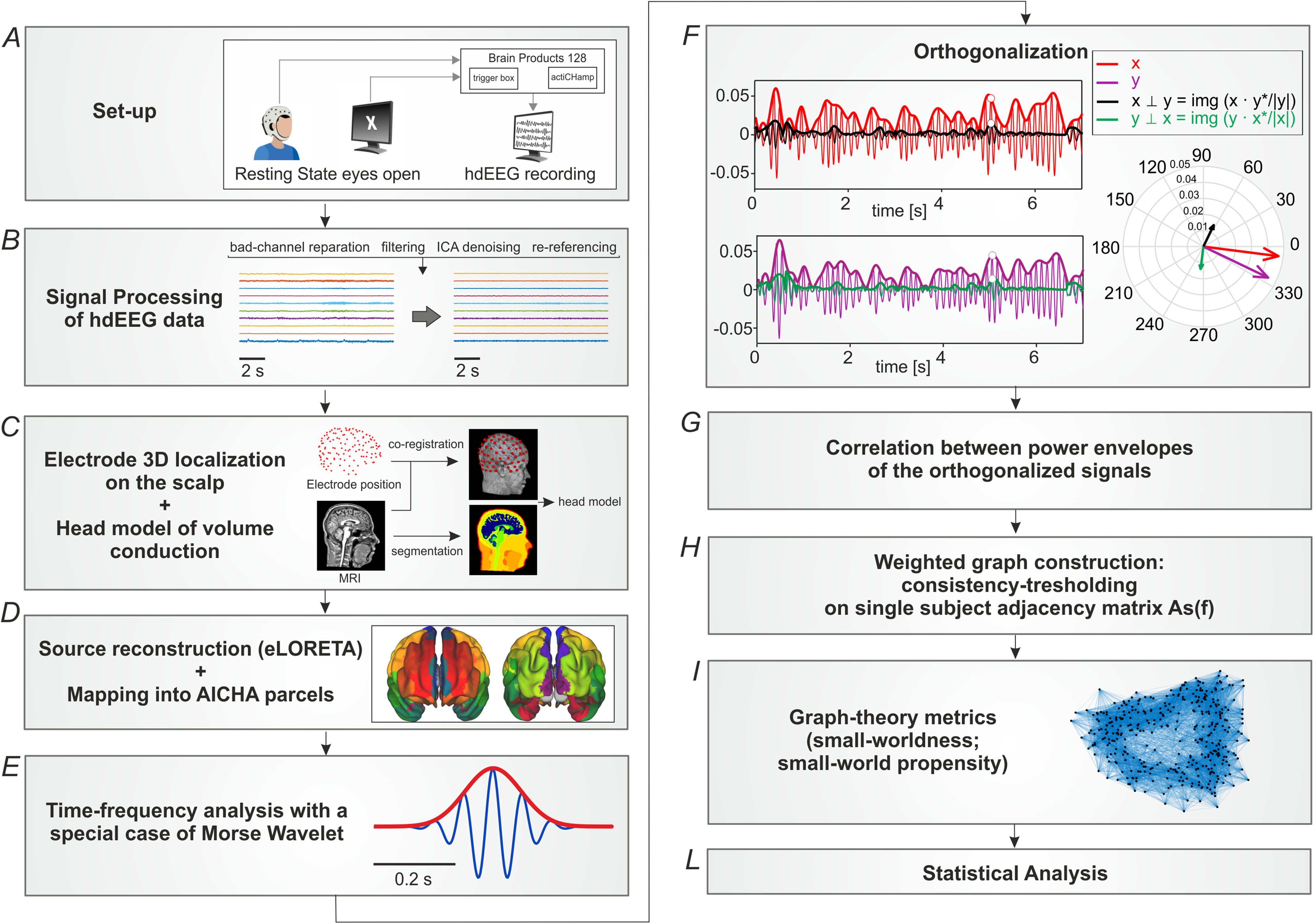
Data analysis pipeline. Overview of the steps to perform the analysis. (A) HdEEG acquisition. (B) Preprocessing of hdEEG data. (C) Head model generation. (D) Source localization. (E) Time-frequency analysis. (F) Orthogonalization. (G) Functional connectivity matrices. (H) Weighted graphs construction. (I) Computation of graph-theory metrics. (L) Statistical analysis, including ANOVA, functional data analysis (FDA) and non-parametric permutation tests.

The adopted analysis pipeline is depicted in Fig. 1.

### A. Participants

We recruited 33 healthy participants (30.4 ± 6.4, mean ± SD years, 18 females). All participants had no previous history of neurological and psychiatric disorders and provided written informed consent prior to participation. A subsample (12 participants, 32.0 ± 8.9, mean ± SD years, 6 females) also underwent a working memory (WM) tasks (see below) for PRE/POST evaluation. The study conformed to the standard of the declaration of Helsinki and was approved by the institutional ethical committee (CER Liguria Ref.1293 of September 12^th^, 2018).

### B. Resting State Recordings and MRI Acquisition

HdEEG was recorded at 1000 Hz sampling frequency from 128 electrodes (actiChamp, Brain Products, Germany; FCz as the physical reference) while participants sat with their eyes open fixating a white cross on a black screen for five minutes. Participants were required to relax as much as possible and to keep fixating the cross (Fig. 1, A). For details about electrooculograms (EOG) and T1-weighted images acquisition parameters, see Suppl. Materials.

### C. Cognitive Task

A subsample of participants performed a WM exercise and we acquired the spontaneous oscillatory activity PRE and POST task execution. The behavioral task consisted of a *n*-back WM task (with *n* = 2, 3) [30], and was implemented in a custom-made Graphical User Interface developed in Matlab (Mathworks, USA). Briefly, a series of pseudo random letters (A, B, C, D, E, F, G, H, I, O) was visually presented in sequence and the participant was required to respond with a button press when the current letter was the same as the letter presented *n* trials earlier. Each letter was displayed for 500 ms with a 2000 ms interval between consecutive stimuli. Each *n*-back sequence comprised of the presentation of 130 letters, 32 of which were stimuli.

### D. Preprocessing of hdEEG Recordings, Head Model and Sources Reconstruction

For hdEEG preprocessing, we followed the same steps described in [1], [2], [11] (Fig. 1, B and Suppl. Materials). To build the volume conductor model, we used T1 weighted structural images (see Fig. 1, C and Suppl. Materials). Then, by combining each head model conductor and the artifacts-free hdEEG signals, we reconstructed brain activity in source space using eLORETA [31], constraining the sources (voxels) with a 6 mm regular grid covering the cerebral gray matter. Then, we mapped individual sources timecourses into *N* = 384 regions of interest (ROIs) of the AICHA atlas [32]. N were the nodes furtherly employed for the graph-theoretical analysis. The activity of each ROI corresponded to the first principal component of the voxels falling within a sphere centered in the ROI center of mass and with 6 mm radius (Fig. 1, D).

### E. Spectral Analysis

We implemented time-frequency analysis by convolving the ROIs signals (${X_i}({t})$, with ${i} = 1..{N}$) with Generalized Morse Wavelets (GMW), described in [33], [34] (Fig. 1, E). This wavelet superfamily guarantees, under certain parametrizations, a strict analytic behavior and therefore is preferable for accurate time-frequency analysis (see Suppl. Materials). We used 23 carrier frequencies, ranging from 2^0.5^ to 2^6^ Hz in quarter steps (${f} = {2^{({0.5:0.25:6})}}$ Hz), to cover a large part of the EEG spectrum with a fine detail.

### F. Weighted Graph Construction

We estimated the FC matrices (384 × 384) between all pairs of nodes and for each carrier frequency, using the methods of power envelope orthogonalization [35], as in previous EEG studies [2], [8]. We removed the coherent zero-lag activity from each pair of ROIs. Then, the pairs of orthogonalized and non-orthogonalized power spectra (X, $\rm {Y}_{\bot \rm {X}}$ and Y, $\rm {X}_{\bot \rm{Y}}$, Fig. 1, F-G) were log-transformed and correlated, using the Pearson coefficient. The resulting correlation was Fisher-transformed to improve Gaussianity (Fig. 1, G). We then obtained one weighted adjacency matrix containing the correlation strength per each frequency and participant (${A_s}(f),s = \text{1}..\text{33}$). Per each frequency, we consistency-thresholded the adjacency matrices according to Roberts *et al*. [36]. We estimated the coefficient of variation (CV) across participants and we thresholded each ${A_s}(f)$ according to CV (from 1% to 100%, the left bound corresponds to sparsely connected matrix, where the survived connections represent the most consistent connections across participants, i.e., with lower CV). Thus, we obtained equally-sparse graphs across participants and we were able to compare graph metrics values over different levels of graph density (Fig. 1, H).

### G. Strength of Functional Connectivity

Prior to graph theoretical analysis, we computed each weighted adjacency matrix [37]. Then, for each participant and for each frequency, we calculated the node strength as the mean of ${A_s}(f)$ columns (obtaining a 384 nodes × 23 frequencies matrix). Thus, we characterized the connectivity strength at node level, frequency by frequency. In order to obtain an overall measure of the average connectivity in each frequency of interest, we further averaged the nodal strength across nodes [37]. Next, we identified those carrier frequencies showing a higher average connectivity when compared with the others. Hence, we performed a repeated measures ANOVA with carrier frequencies as within-participants factor as in [38]. We tested the sphericity assumption with Mauchly's test, and applied the Greenhouse-Geisser correction, if rejected. Then, we implemented Tukey HSD post-hoc test to investigate significant differences among the 23 frequencies. We further applied Bonferroni correction for multiple comparisons (*p* < 0.0022, after correcting for the number of compared carrier frequencies).

### H. Graph Theoretical Analysis: Small-World Propensity

SWP indicates the network propensity to show small-world architecture (https://complexsystemsupenn.com/codedata) [19]. SWP takes into account the discrepancy of the observed ${A_s}(f)$ weighted clustering coefficient $({{C_{\rm{obs}}}(f)})$ and shortest path length $({{L_{\rm{obs}}}(f)})$ from equivalent (i.e., with the same strength distribution, number of nodes and edges density) lattice (${C_{\rm{latt}}}(f)$ and ${L_{\rm{latt}}}(f)$) and random (${C_{\rm{rand}}}(f)$ and ${L_{\rm{rand}}}(f)$) networks (ΔC and ΔL, respectively). Hence, SWP is defined, per each carrier frequency, as:

\begin{equation*}
SWP\left(f \right) = 1 - \sqrt {0.5 \times \left({\Delta C{{\left(f \right)}^2} + \Delta L{{\left(f \right)}^2}} \right)} 
\end{equation*}

Where:

\begin{align*}
\Delta C\left(f \right)& = \left({{C_{\rm{latt}}}\left(f \right) - {C_{\rm{obs}}}\left(f \right)} \right)/\left({{C_{\rm{latt}}}\left(f \right) - {C_{\rm{rand}}}\left(f \right)} \right)\\
\Delta L\left(f \right)& = \left({{L_{\rm{obs}}}\left(f \right) - {C_{\rm{rand}}}\left(f \right)} \right)/\left({{L_{\rm{latt}}}\left(f \right) - {L_{\rm{rand}}}\left(f \right)} \right)
\end{align*}

For the weighted clustering coefficient we followed the definition of Onnela *et al*. [39] and for the shortest path length we employed the Matlab function *graphallshortestpaths.m* [40]. The small-world regimen is reached when the SWP exceeds a value of 0.6 [19]. Importantly, while the classic small-worldness (σ) index [41] accounts only for deviation from a random-equivalent model, the SWP index considers the contribution to deviation from both lattice and random model. Moreover, different to σ, SWP does not depend on graph density [19]. We computed both SWP and σ for the entire RS dataset (Fig. 1, I), and, for those participants who performed the cognitive exercise, we evaluated SWP changes PRE/POST WM task.

### I. FDA Statistical Analysis

To characterize the potential frequency-specific behavior of the SWP index, we employed the Functional Data Analysis (FDA), as in a previous work [37], which provides a statistical framework to compare graph theoretical metrics (Fig. 1, L). For further information about FDA analysis, see Suppl. Materials.

## III. Results

In this work, we characterized the connectivity strengths and the small-world frequency-specific properties during RS and after a WM task. When presenting the results, we associate the carrier frequencies to the corresponding frequency bands, i.e., we localized the carriers into the main EEG spectral bands, here defined as: delta (δ, 1-4 Hz), theta (θ, 4-8 Hz), alpha (α, 8-13 Hz), beta (β, 13-30 Hz), gamma (γ, 30-80 Hz), as in [42].

### A. Frequency-Specific Functional Connectivity

**Fig. 2.**
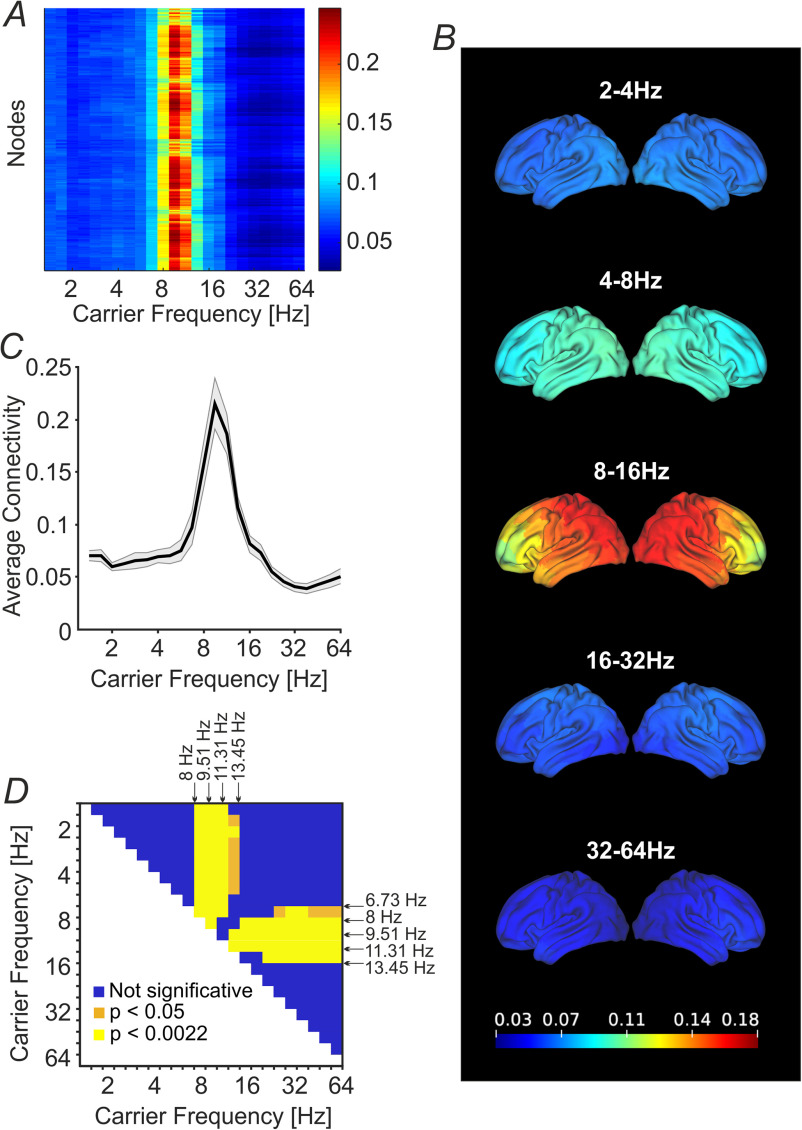
Node strength and average connectivity. (A) Node strength across participants in each wavelet carrier frequency (${2^{({\text{0.5:0.25:6}})}}$Hz), the X-axis is in ${\rm{log}_2}$ scale. (B) Node strength frequency specificity overlaid onto the T1-weighted template. For visualization purpose, we further averaged the strength values of the quadruplets between the integer carrier frequencies, obtaining the bands: 2-4 Hz, 4-8 Hz, 8-16 Hz, 16-32 Hz, 32-64 Hz. The colormap is kept fixed to the minimum and maximum values across the bands. See Fig. S1 in the Suppl. Materials where the colormap is customized between minimum and maximum values in each frequency band of interest. (C) The average connectivity is frequency-dependent. Black line and gray shaded-areas indicate mean ± SE across nodes and participants. The X-axis is in ${\rm{log}_2}$ scale. (D) Results of the Post-hoc Tukey's HSD test on carrier frequencies. For each couple of frequencies, the lighter elements show a statistically significant difference (yellow: Bonferroni-corrected for multiple comparisons (*p* < 0.0022), orange: uncorrected (*p* < 0.05)). Dark blue elements indicate those frequencies that did not significantly differ among each other. X-axis and Y-axis are in ${\rm{log}_2}$ scale.

Fig. 2, A represents the node strengths across participants: a peak emerges in the carrier frequency centered in the EEG high theta band (*f* = 6.73 Hz), reaching a maximum in the alpha band (*f* = 8, 9.51, 11.31 Hz) and decreasing in low beta-centered carrier frequencies (*f* = 13.45, 16 Hz). We overlaid nodal strengths values onto the T1-weighted template showing the frequency-specific behavior of nodal strengths across frequency bands (Fig. 2, B). The same frequency-dependent pattern emerged for the average connectivity values as depicted in Fig. 2, C (ANOVA frequency factor F(22,704) = 27.1, *p* < 0.0001, ε = 0.12, Greenhouse-Geisser corrected). The post-hoc statistical analysis (see Fig. 2, D) revealed a significant increase of average connectivity mainly for the carrier frequencies localized in the alpha band (*f* = 8, 9.51, 11.31, yellow elements in Fig. 2, D) when compared with the other bands. In addition, the low beta band carrier frequency (*f* = 13.45 Hz) was statistically higher than high beta and gamma waves (yellow elements, in Fig. 2, D), as well as higher than delta and theta bands, but to a lesser extent (yellow and orange elements, in Fig. 2, D). High delta carrier frequency (*f* = 6.73 Hz) was significantly greater than high beta and gamma bands (orange elements, Fig. 2, D). Finally, for all the remaining comparisons, the average connectivity had low intensity and it was not significantly different across these frequencies (blue elements in Fig. 2, D). See Table S2 for an overview of the mean ± SD average connectivity values across carrier frequencies.

### B. Small-Worldness Frequency-Specific Behavior

**Fig. 3.**
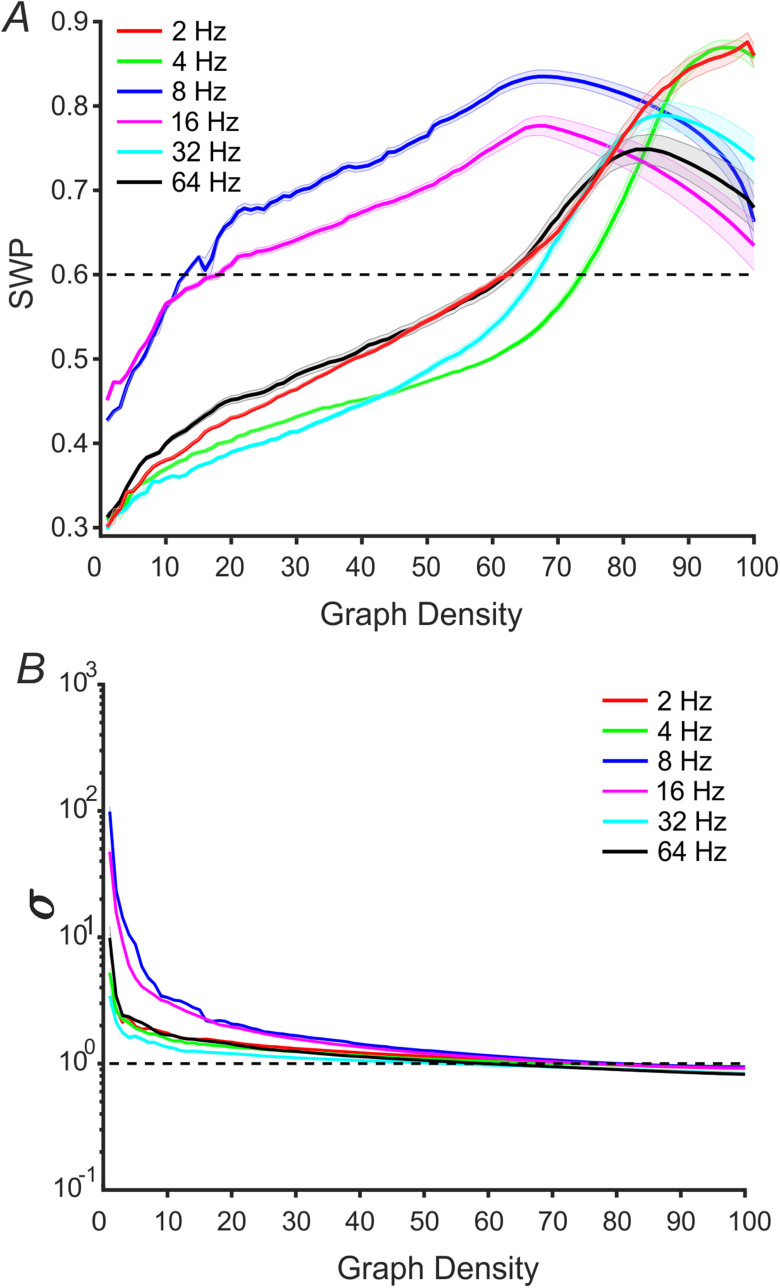
Small-worldness across graph density. (A) SWP vs. graph density curves. Black dotted line indicates the 0.6 threshold for small-worldness. (B) σ vs. graph density curves. Black dotted lines show the 1 threshold for small-worldness. Y-axis is in $\rm{log}{_{10}}$ scale. In both panels, each color identifies a different carrier frequency (${f} = 2,4,8,16,32,64$ Hz). Lines and shaded areas show, mean ± SE, respectively.

As for the SWP index during RS (Fig. 3, A), we found a statistically significant difference when comparing the six integer carrier frequencies among each other (FDA analysis, *p* < 0.0001 in all the comparisons). The averaged curves for 8 and 16 Hz vs. graph densities (blue and magenta lines, respectively), indicated the highest small-world topological organization across all the analyzed frequencies, up to graph density of ∼75%. The 8 Hz frequency entered the small-world regimen (SWP > 0.6) at 13% of graph density while the 16 Hz reached small-worldness at 19%. Both frequencies maintained small-worldness for all other ranges of graph densities. The remaining curves (2, 4, 32 and 64 Hz) showed similar, moderate SWP values (less than 0.6, but higher than 0.4 [16]) from low graph density ranging from 10% to about 60% graph density. For very low graph density (less than 10%), these four averaged curves showed the smallest SWP values (lowest bound is ∼0.3). Instead, after the ∼60% graph density, we had the onset of small-world regimen and SWP remained above the 0.6 threshold until the right graph density limit. For higher graph density (>∼75%) SWP values showed the greatest within- and between- variability for all the considered frequencies.

As for the small-world index (σ), all the selected frequencies statistically differed from each other (FDA analysis, *p* < 0.0001, for all comparisons, see Fig. 3, B). Likewise SWP index, σ also exhibited the highest small-worldness intensity in the 8 and 16 Hz carrier frequencies (Fig. 3, B blue and magenta lines). Small-worldness averaged curves showed an exponential decay that retains small-world property (above 1) respectively until 80% and 76% graph density, for 8 and 16 Hz. The two curves achieved the highest small-worldness for low graph density (from 1% to ∼5%). On the other hand, the remaining frequencies (i.e., 2, 4, 32 and 64 Hz) showed the same exponential trend, but with different constants decay. The decay caused small-worldness to disappear within the range of 55%-60% graph density, depending on the carrier frequency.

### C. Modulation of Frequency-Specific Properties Induced by Task Performance

**Fig. 4.**
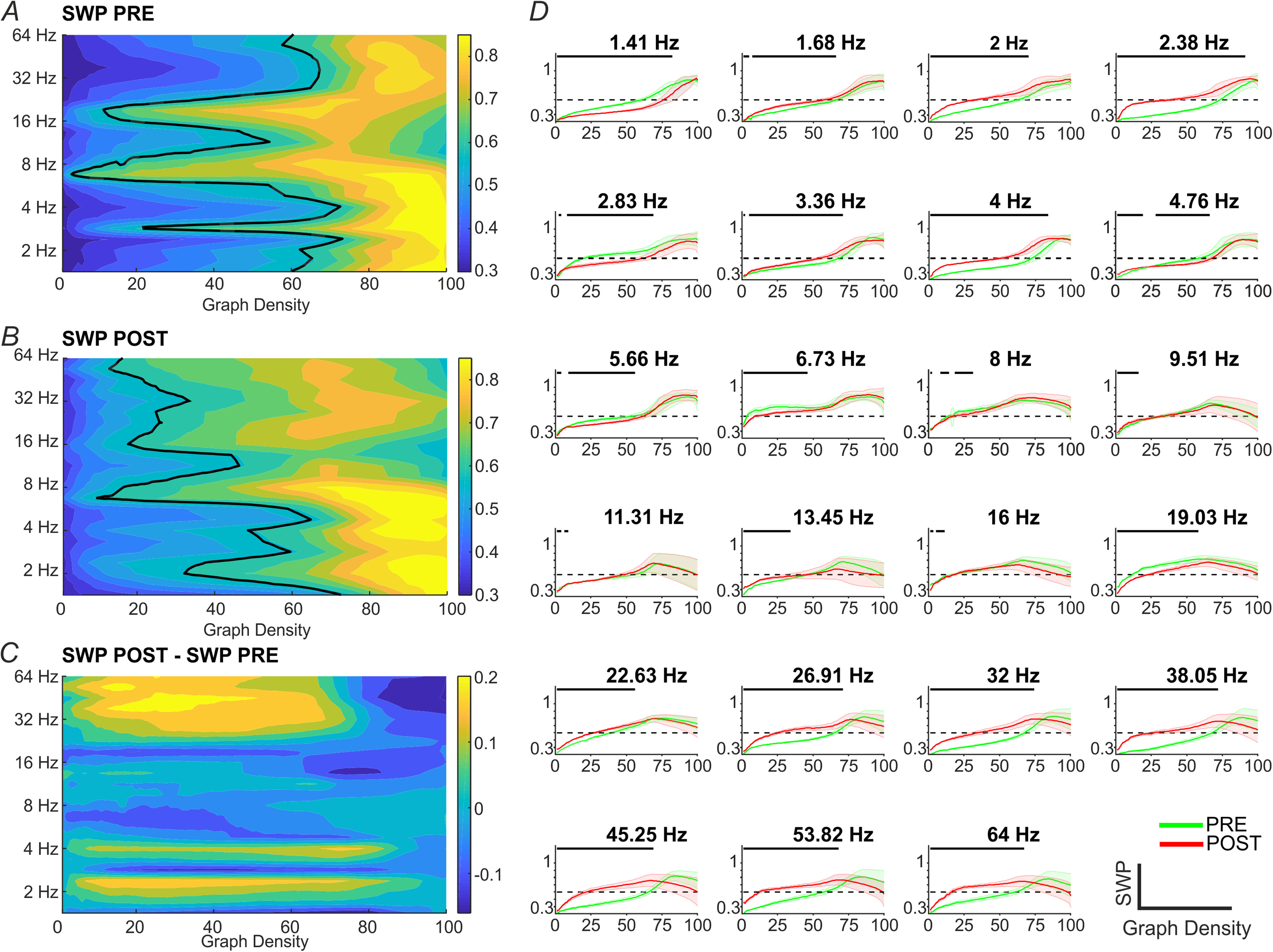
Modulation of SWP by WM task. (A and B) SWP values during RS in the PRE (A) and POST (B) WM task as a function of the carrier frequencies (Y-axis, in ${\rm{log}_2}$ scale) and of the graph density (X-axis). Black contour lines indicate SWP threshold for small-worldness (SWP = 0.6). (C) SWP systematic difference between POST and PRE condition, note the different colorbar range with respect to panels A and B. (D) PRE and POST SWP vs. graph density curves in each of the 23 carrier frequencies. Continuous green and red lines (mean values across participants) and shaded areas (SD values across participants) indicate PRE and POST conditions, respectively. Black bars at the top of each panel, represent the graph densities for which the p-value of a paired t-test indicates a significant difference between the PRE and POST conditions (*p* < 0.0022, Bonferroni corrected).

Fig. 4, A-B, show respectively SWP values vs. both frequencies and graph density in PRE and POST conditions, computed for the subsample of participants who underwent the WM task. The difference between the two conditions is highlighted in Fig. 4, C which reports the systematic difference between the SWP values POST and PRE WM task. When looking at the single carrier frequency, we observed a change in the shape of the averaged SWP vs. graph density curves in the PRE and POST condition (see, Fig. 4, D) in a subset of frequencies. Indeed, the SWP increased in the POST condition, when compared with the PRE condition (FDA analysis, *p* < 0.0001) for most frequencies belonging to delta rhythm (1.68, 2, 2.38, 3.36, 4 Hz, see Fig. S2, black permutation distributions) as well as for most frequencies in the high beta and gamma bands (19, 26.91, 32, 38.05, 45.25, 53.82 and 64 Hz, see Fig. S2). Instead, SWP during PRE was greater than during POST for 1.41, 2.83 and 19.03 Hz frequencies (*p* < 0.0001). The other carrier frequencies did not show a statistically significant change in the SWP values (see light-gray distributions in Fig. S2).

## IV. Discussion

With this study, we characterized the frequency specificity of the connectivity strength and the small-world properties of RS activity, recorded with hdEEG.

### A. Alpha and Low Beta Band Increase of the Connectivity Strength

This study extends, with the use of hdEEG and weighted networks analysis, previous findings [2], [8], [27] about whole-brain FC structure frequency specificity, showing alpha and low beta band increase in nodal strengths and average connectivity. In these carrier frequencies (i.e., from 8 Hz to 16 Hz), a spatially distributed gradient (in posterior-anterior direction) emerged from high to low nodal strength values (see Fig. 2, B and Fig. S1). A similar spatially distributed FC pattern was reported in [35].

### B. SWP and σ Show Different Small-Worldness Information

We found that the 8 Hz and 16 Hz carrier frequencies, which showed the highest connectivity strengths, also exhibited the strongest small-world architecture for both indices (blue and magenta curves, Fig. 3). This result suggests that for RS electrophysiological activity, the optimization of information transfer occurs between alpha and low beta band. However, SWP and σ show different behaviors when considered across graph densities. Indeed, 8 and 16 Hz, for low graph density (less than ∼20% of graph density) showed small-worldness regimen only when considering the σ index, but not SWP, that, by contrast, indicate mild (range: 0.3-0.4) to moderate (<0.4) small-world organization [16]. Moreover, the SWP for the remaining frequencies (2, 4, 32, and 64 Hz) was below the 0.6 threshold until ∼60%, while σ indicated small-worldness architecture (above 1) until ∼60% graph density. However, σ suffers from a bias due to graph density, suggesting that SWP is more suited than σ to characterize the small-world topology [19]. Moreover, by increasing the graph density (after 60-65%, depending on the considered frequencies), σ fell below the σ = 1 threshold. With respect to this, we underline that according to Muldoon *et al*. [19], caution must be taken while trying to impose small-world formalism [17] when the graph is dense. With respect to this issue, our data indicates that for high graph density, SWP values resulted in more variability across participants (i.e., SE increase, see Fig. 3, A). Moreover, SWP values of 8 and 16 Hz frequencies fell below the SWP of other frequencies (Fig. 3, A), but still maintained small worldness regimen (≥ 0.6). Frequency specificity was reliably investigated using the FDA statistical approach, which by definition takes into account the entire graph density range for both SWP and σ. Indeed, despite the influence that different graph densities may have on the estimated graph-theoretical metrics, we believe that analyzing fully dense (no thresholding applied) FC networks could be of interest in other applications, such as the detection of hidden communities’ structures in weighted networks. In fact, thresholding can be detrimental in order to learn the underlying community structure [43].

### C. Modulation of Small-Worldness Induced by Cognitive Load

We found a significant increase of SWP in high beta and gamma bands (≥26.91 Hz) and unchanged SWP values in alpha band and low beta. Previous MEG and EEG studies demonstrated that theta and gamma waves are specifically related to WM processing [44], moreover, a gamma modulation has been associated to higher cognitive load [45]. During WM task, these bands reflect a consolidation of the small-world architecture (i.e., SWP increases), and we speculate that during the following RS acquisition (POST condition) the increased small-world organization persists, thus still observing SWP values higher than the PRE condition. We did not find significant difference between conditions in the alpha and early beta oscillations. Indeed, although these bands are also partially involved during WM tasks [44], they mainly constitute the predominant rhythms during RS [2], [27] and therefore also in the POST condition, so they are plausibly stronger than all the other rhythms.

## V. Conclusions

In this work, we investigated the frequency specificity of RS FC by using nodal connectivity strength, average connectivity and small-world parameters (SWP and σ). We also computed SWP as a sensitive biomarker of frequency-dependent spontaneous activity alterations, following a WM task. Importantly, the influence of task execution on RS activity is relevant in many application domains, such as sensorimotor rehabilitation [46], [47]. Within this framework, our long-term goal is to validate the effectiveness of a neurorehabilitation intervention, as with robot-assisted training, through the evaluation of RS-FC [48]. RS has indeed the advantage of being well suited also for highly injured patients [49]. Moreover, it is not linked to task-related parameters, is less affected by motion artifacts, that, in case of EEG studies, may decrease the signal to noise ratio, and is a valid tool to explore the retention of the rehabilitation program on a long-term perspective.

In conclusion, here we demonstrated that it is possible to estimate SWP properties from FC weighted network with hdEEG data, and that this metric is a valid tool to unravel the frequency-specific signatures of RS activity.

## Supplementary Materials

The Supplementary Information is described in more detail in the Supplementary Materials file, which includes the following Sections: Methods (Electrooculograms, Electrode localization and MRI acquisitions; Pre-processing of hdEEG recordings; Head model and source reconstruction; Generalized Morse Wavelet; Functional Data Analysis); Tables (Supplementary Tables TS1: T1-weighted acquisition parameters for the participants of the study, and TS2: Average connectivity values across participants in each carrier frequency); Figures (Fig. S1: Node strength frequency-specificity overlaid onto the T1-weighted template, and Fig. S2: Permutation distributions related to FDA-statistical testing between PRE and POST working memory task.); additional References.


