# Supplementary material for: Small-World Propensity Reveals the Frequency Specificity of Resting State Networks
Source: IEEE Open J Eng Med Biol. 2020 Feb 14;1:57–64. doi: 10.1109/OJEMB.2020.2965323 (PMC8979624; doi:10.1109/OJEMB.2020.2965323)
Supplement: Supplementary file 1 [file supp1-2965323.pdf]

Supplementary Materials

Small-World Propensity Reveals the Frequency Specificity of Resting State Networks

Riccardo Iandolo[1], *Member, IEEE*, Marianna Semprini[1], *Member, IEEE*, Stefano Buccelli[1-2], *Member, IEEE*, , Federico Barban[1-2], *Member, IEEE*, Mingqi Zhao[3], Jessica Samogin[3], Gaia Bonassi[4], Laura Avanzino[4-5], Dante Mantini[3-6] and Michela Chiappalone[1*], *Member, IEEE*

I. Methods

A. Electrooculograms, Electrode localization and MRI acquisitions

The horizontal and vertical electro-oculograms (EOG) were collected from the right eye for further identification and removal of ocular-related artifacts. Prior to resting-state hdEEG recordings, we collected the three-dimensional locations of the 128 electrodes using either infrared color-enhanced 3D scanner [1] or Xensor digitizer (ANT Neuro, The Netherlands). Then, we used the software described in [1] to identify the electrode's coordinates on the scalp of each individual. To build each individual high-resolution head model, participants previously underwent T1-weighted image acquisition using either a 3 T or 1.5 T MRI scanners (see Table S1).

B. Pre-processing of hdEEG recordings

For hdEEG preprocessing we followed the steps presented in previous works [2, 3] (Fig.1, B). Briefly, we first attenuated power noise by using a notch filter centered at 50 Hz. Secondly, we performed an automatic procedure to identify channels with low signal to noise ratio. Then, we combined information from two channel-specific parameters: i) the minimum Pearson correlation between a channel against the remaining channels in a frequency band of interest (0.5-100 Hz); ii) the noise variance that we defined in a band where the EEG information is negligible (200-250 Hz). We defined a channel as "bad", whenever one of the two parameters described above were outliers as compared to the total distribution of values. We interpolated the identified bad channels with the information of the neighboring channels, using Field Trip (http://www.fieldtriptoolbox.org/). Then, hdEEG signals were band pass filtered (0.5-100 Hz) with a FIR zero-phase distortion filter and downsampled to 250 Hz. To further reduce noise in our data, we employed the fast-ICA algorithm to identify independent components related to ocular and movement artifacts. To classify the ocular artifacts we used the following parameters: i) Pearson correlation between the power of the independent components and the vertical and horizontal EOG; ii) the coefficient of determination obtained by fitting the IC spectrum with a 1/f function. We classified the IC as ocular artifacts if at least one of the two parameters was above a pre-defined threshold (0.2 and 0.5, as in previous studies [2, 4]). Finally, for movement-related artifacts, we used the kurtosis of the independent component (we considered a noisy independent component if the kurtosis exceeded a value of 20 [2]). We re-referenced the artifacts-free signals with the average reference approach [5].

C. Head model and source reconstruction

We used T1-weighted structural images to build the volume conductor model (see Fig.1, C). According to previous studies [2, 3], we assigned a conductivity value to 12 tissues (skin, eyes, muscle, fat, spongy bone, compact bone, gray matter, cerebellar gray matter, white matter, cerebellar white matter, cerebrospinal fluid and brainstem). Then, to segment the tissues, we warped the MNI template to each individual subject space using the normalization tool of SPM12 (http://www.fil.ion.ucl.ac.uk/spm/software/spm12), as reported in Liu et al [2]. We projected the electrode positions onto the individual T1-weighted space. We approximated the volume conduction model using finite element method (FEM) and, to estimate the relationship between the scalp potentials and the sources dipoles, we employed the Simbio FEM method (https://www.mrt.uni-jena.de/simbio/).

D. Generalized Morse Wavelet

The GMW is defined, in the frequency domain, as:

$$\psi_{\beta,\gamma}(f) = a_{\beta,\gamma} f^{\beta} e^{-f^{\gamma}}$$

Where, $a_{\beta,\gamma} = 2(e\,\gamma/\beta)^{\beta/\gamma}$ is a normalizing constant, f are the carrier frequencies of the wavelet and β and γ are the two parameters controlling the wavelet shape. As suggested in [6], a choice of γ = 3, guarantees the most symmetric, most nearly Gaussian, and generally most time-frequency concentrated member (thanks to the minimum Heisenberg area) of the GMW superfamily. In this work, we thus set γ = 3.00 and β = 11.33 to capture the essential idea of the widely used Morlet wavelet [7, 8], while avoiding aliasing for specific parameter choices [6]. We employed the Matlab version of the Jlab toolbox (freely available online: http://www.jmlilly.net/jmlsoft.html).

E. Functional Data Analysis

Functional Data Analysis (FDA) is a valid statistical tool for our goal, because it allows for between-conditions comparison and is independent from the threshold choice (i.e. graph density), thus including the entire range of graph density [9]. Briefly, we obtained the averaged (across participants) SWP vs. graph density curve for six carrier frequencies (f = 2, 4, 8, 16, 32, 64 Hz). Then, we compared each couple of frequencies,

obtaining the averaged curves X_{f1} and X_{f2} across participants (where f_1 and f_2 is a couple of the six frequencies, with $f_1 \neq f_2$) and we calculated the area between them ($A_{f1-f2,real}$). We performed a non-parametric permutation test to reveal statistically significant differences between each couple of curves. We randomly assigned the 66 curves (2 frequencies × 33 participants) to either one of the two frequencies taken into consideration. We calculated the area between these averaged curves and we iterated this entire procedure 10’000 times, obtaining a null permutation distribution ($A_{f1-f2,perm}$) against which we compared the real area. We defined as empirical p-value the ratio between the number of times where $A_{f1-f2,perm}$ was greater than $A_{f1-f2,real}$ divided by the total number of permutations. Similarly, we performed FDA analysis for the working memory task dataset. In this case, we compared, for all 23 carrier frequencies, the averaged across participants SWP curves $X_{PRE}(f)$ and $X_{POST}(f)$ and we created the permutation distribution by reassigning the SWP curves of the PRE and POST conditions without replacement. We computed the empirical p-values as in the previous permutation test.

II. TABLES

Table S1. T1-weighted acquisition parameters for the participants of the study, who underwent T1-weighted using either a 3T or 1.5 T scanner.

**Number of participants**	22	7	4
**MRI scanner**	3T Achieva (Philips Medical System, The Netherlands)	3 T MAGNETOM Prisma (Siemens AG, Healthcare Sector, Germany)	1.5 T Signa Excite (General Electric Healthcare, USA)
**T1-weighted sequence name**	Magnetization Prepared Rapid Acquisition Gradient Echo (MPRAGE)	Magnetization Prepared Rapid Acquisition Gradient Echo (MPRAGE)	Fast Spoiled Gradient Echo (SPGR)
**Coil**	32-channel head coil	32-channel head coil	8-channels phased array head coil
**Voxel Size**	0.98×0.98×1.2 mm^3	1×1×1 mm^3	1×1×1 mm^3
**TR/TE**	9.6/4.6 ms	2.3/2.96 ms	11.8/5.18 ms
**Field of View (F.O.V)**	250x250 mm^2	256×256 mm^2	256×256 mm^2

Table S2. Average connectivity values across participants in each carrier frequency. Data are reported as mean ± SD values of average connectivity in each of the twenty-three carrier frequencies

	Mean ± SD
1.41 Hz	0.070604 ± 0.027949
1.68 Hz	0.070184 ± 0.031342
2 Hz	0.060131 ± 0.021806
2.38 Hz	0.062686 ± 0.031226
2.83 Hz	0.065567 ± 0.045826
3.36 Hz	0.066657 ± 0.039863
4 Hz	0.069618 ± 0.034671
4.76 Hz	0.070299 ± 0.043268
5.66 Hz	0.075459 ± 0.058049
6.73 Hz	0.096566 ± 0.087903
8 Hz	0.157838 ± 0.126582
9.51 Hz	0.215224 ± 0.137532
11.31 Hz	0.186046 ± 0.108915
13.45 Hz	0.115930 ± 0.058217
16 Hz	0.081514 ± 0.044912
19.03 Hz	0.073346 ± 0.043180
22.63 Hz	0.054945 ± 0.029200
26.91 Hz	0.046133 ± 0.027674
32 Hz	0.040597 ± 0.024060
38.05 Hz	0.039110 ± 0.025210
45.25 Hz	0.042566 ± 0.029534
53.82 Hz	0.046528 ± 0.035692
64 Hz	0.050638 ± 0.040119

III. Figures

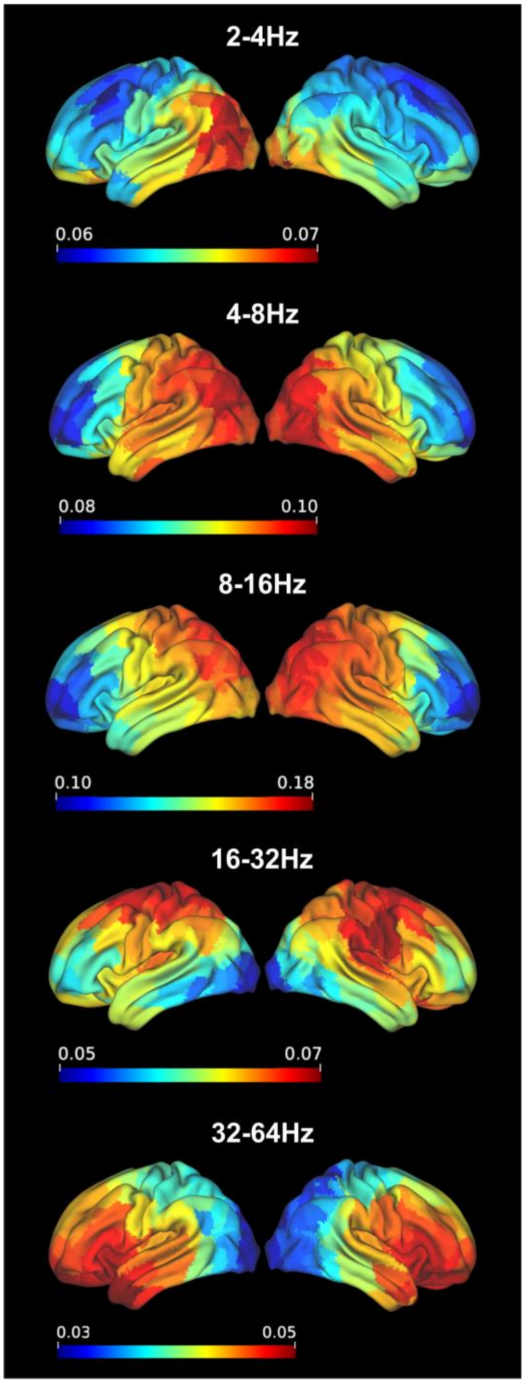

Fig. S1. Node strength frequency-specificity overlaid onto the T1-weighted template. For visualization purpose, we further averaged, as in the main manuscript, the average connectivity values of the quadruplets between the integer carrier frequencies, obtaining the bands: 2-4 Hz, 4-8 Hz, 8-16 Hz, 16-32 Hz, 32-64 Hz. The colormap is customized between the minimum and maximum values of each frequency band.

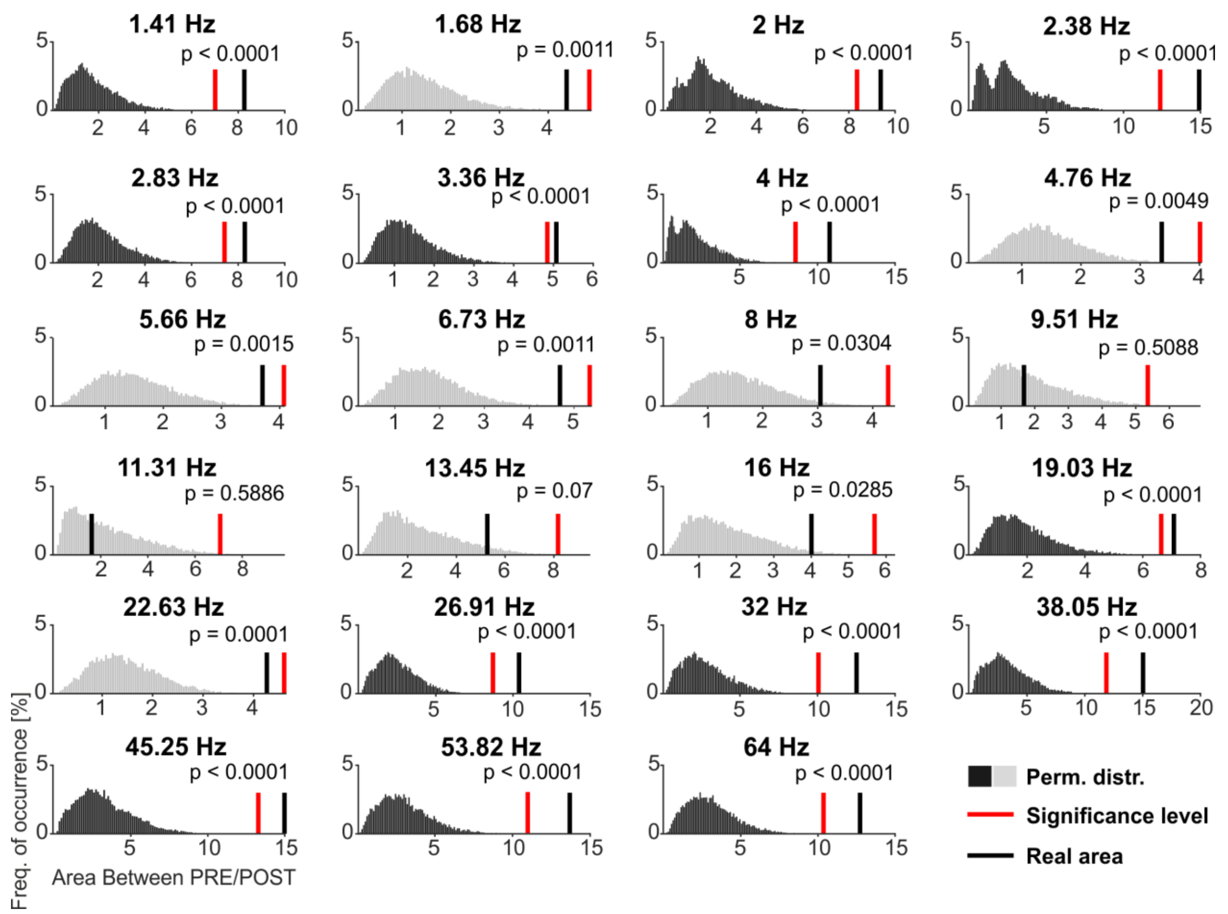

Fig S2. Permutation distributions related to FDA-statistical testing between PRE and POST working memory task. Black distributions indicate carrier frequencies for which the permutation test was statistically significant. Light-gray distributions show absence of significant difference. Red bars indicate empirical p-value (see methods). Black bars indicate the area (on the X-axis) underlying the PRE and POST curve.

IV. References

[1] G. A. Taberna, R. Guarnieri, and D. Mantini, "SPOT3D: Spatial positioning toolbox for head markers using 3D scans," *Scientific reports,* vol. 9, no. 1, pp. 1-9, 2019.

[2] Q. Liu, S. Farahibozorg, C. Porcaro, N. Wenderoth, and D. Mantini, "Detecting large-scale networks in the human brain using high-density electroencephalography," *Human brain mapping,* vol. 38, no. 9, pp. 4631-4643, 2017.

[3] J. Samogin, Q. Liu, M. Marino, N. Wenderoth, and D. Mantini, "Shared and connection-specific intrinsic interactions in the default mode network," *Neuroimage,* vol. 200, pp. 474-481, 2019.

[4] F. de Pasquale *et al.*, "Temporal dynamics of spontaneous MEG activity in brain networks," *Proc Natl Acad Sci U S A,* vol. 107, no. 13, pp. 6040-5, Mar 30 2010.

[5] Q. Y. Liu, J. H. Balsters, M. Baechinger, O. van der Groen, N. Wenderoth, and D. Mantini, "Estimating a neutral reference for electroencephalographic recordings: the importance of using a high-density montage and a realistic head model," (in English), *Journal of Neural Engineering,* vol. 12, no. 5, Oct 2015.

[6] J. M. Lilly and S. C. Olhede, "Generalized Morse Wavelets as a Superfamily of Analytic Wavelets," (in English), *Ieee Transactions on Signal Processing,* vol. 60, no. 11, pp. 6036-6041, Nov 2012.

[7] C. Tallon-Baudry, O. Bertrand, C. Delpuech, and J. Pernier, "Stimulus specificity of phase-locked and non-phase-locked 40 Hz visual responses in human," *J Neurosci,* vol. 16, no. 13, pp. 4240-9, Jul 1 1996.

[8] J. F. Hipp, D. J. Hawellek, M. Corbetta, M. Siegel, and A. K. Engel, "Large-scale cortical correlation structure of spontaneous oscillatory activity," *Nat Neurosci,* vol. 15, no. 6, pp. 884-90, Jun 2012.

[9] D. S. Bassett, B. G. Nelson, B. A. Mueller, J. Camchong, and K. O. Lim, "Altered resting state complexity in schizophrenia," *Neuroimage,* vol. 59, no. 3, pp. 2196-207, Feb 1 2012.
